# Needle Embolism to the Heart: A Case Report and Review

**DOI:** 10.7759/cureus.14469

**Published:** 2021-04-13

**Authors:** Scott Hatcherson, Vikramaditya S Venkata, Surya Aedma, Nikhil Nalluri, Mini L Sivadasan

**Affiliations:** 1 Graduate Medical Education, Methodist Health System, Dallas, USA; 2 Internal Medicine, Cheshire Medical Center, Dartmouth-Hitchcock, Keene, USA; 3 Internal Medicine, Carle Foundation Hospital, Urbana, USA; 4 Cardiology, Staten Island University Hospital / Northwell Health, Staten Island, USA; 5 Cardiothoracic Surgery, Methodist Health System, Dallas, USA

**Keywords:** foreign body embolism, needle in pericardium, needle causing pericardial effusion, right ventricular foreign body, iv needle embolism

## Abstract

Needle embolisms in the heart are quite rare, and their management is largely based on clinical experience. We describe a patient with chest pain and shortness of breath, whose electrocardiogram revealed subtle inferolateral ST segment elevations. The patient was found to have a bloody pericardial effusion causing tamponade from a long-ago injected needle. Removal of a needle is a complicated decision, that should be done in a multi-disciplinary fashion to minimize complications. Removal may not always be necessary if the needle is in a stable position and not in danger of migration.

## Introduction

Foreign bodies in the heart are very rare, but needle embolisms in the myocardium and pericardium are even rarer. The rarity of this condition and the lack of formal guidelines demonstrate the importance of having high clinical suspicion in the case of an intravenous drug user (IVDU) presenting with chest pain. This article was previously presented as a meeting abstract at the 2020 Texas Chapter of the American College of Physicians Annual Meeting on November 6-8, 2020.

## Case presentation

History of presentation

A 28-year-old male presented with acute pressure-like chest pain and dyspnea that had started suddenly the night prior to arrival after using crystal methamphetamine. He had no aggravating or alleviating factors and no other associated symptoms. On physical examination, the patient was afebrile at 36.5°C and tachycardic in the range 115-125 beats per minute. His respiratory rate was 18-30 breaths per minute, and his blood pressure was ranging wildly from 131/117 at its highest to below 90/60. The patient was anxious, in acute distress, and diaphoretic with distant heart sounds and rapid faint distal pulses.

Past medical history

The patient had a known history of metabolic syndrome, untreated hepatitis C, and intravenous drug abuse with methamphetamines and heroin. He had had two needles break off in his right arm two years prior to the current event. He was taking suboxone for drug abuse. He had no prior surgeries or other known medical conditions.

Differential diagnosis

This patient’s angina and dyspnea were both most likely a result of a needle traumatizing the pericardial sac, leading to hemorrhagic pericardial effusion and tamponade. Other possible etiologies included acute coronary syndrome, pericarditis, and myocarditis.

Investigations

**Figure 1:**
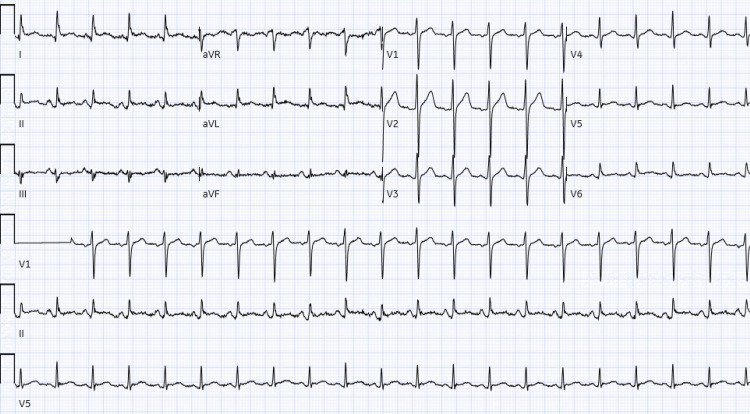
Electrocardiogram on arrival

**Figure 2:**
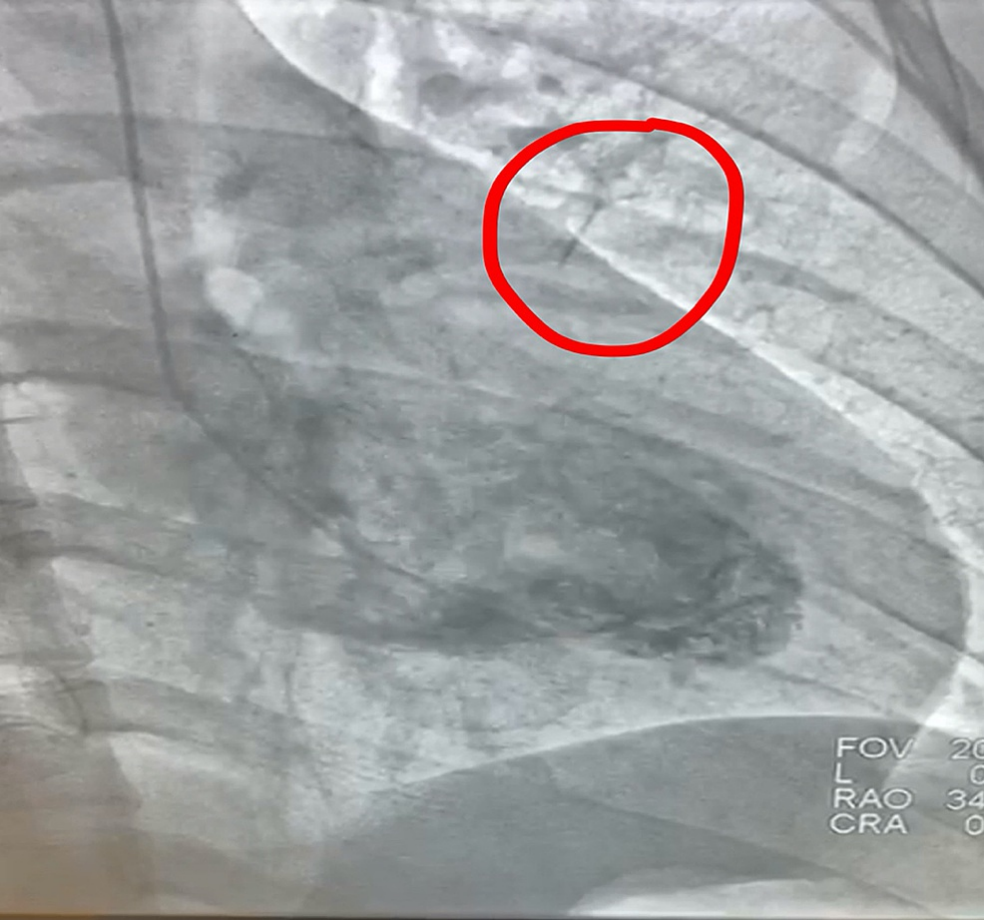
Angiography showing presence of a needle

**Figure 3:**
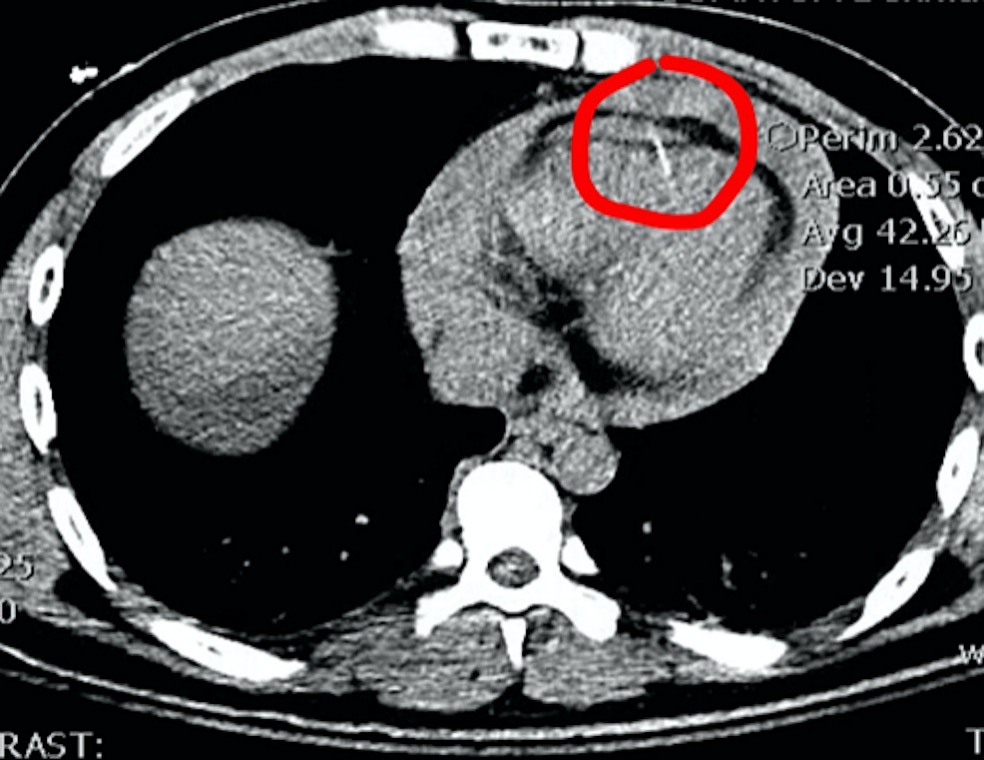
Computerized tomography showing presence of needle

The initial troponin was normal (<0.012 ng/mL), and the initial chest X-ray was unrevealing. An electrocardiogram in the emergency department demonstrated subtle ST elevations in the inferolateral leads, which led to a concern for ST-elevation myocardial infarction (STEMI). A right and left coronary angiogram and left ventriculogram with multiple views were therefore performed (Figures 1-2). These demonstrated normal coronary anatomy with mild atherosclerosis, an ejection fraction of 55%, and mild inferior hypokinesis. It appeared as if there was a foreign body inside the heart, so a stat computed tomography (CT) scan was ordered to rule this out and an echocardiogram obtained to eliminate the possibility of tamponade (Figure 3). The CT scan confirmed the presence of a needle in the right ventricle, and the echocardiogram increased the suspicion of tamponade, showing partial right ventricle diastolic collapse in a hemodynamically unstable patient.

Management

**Figure 4:**
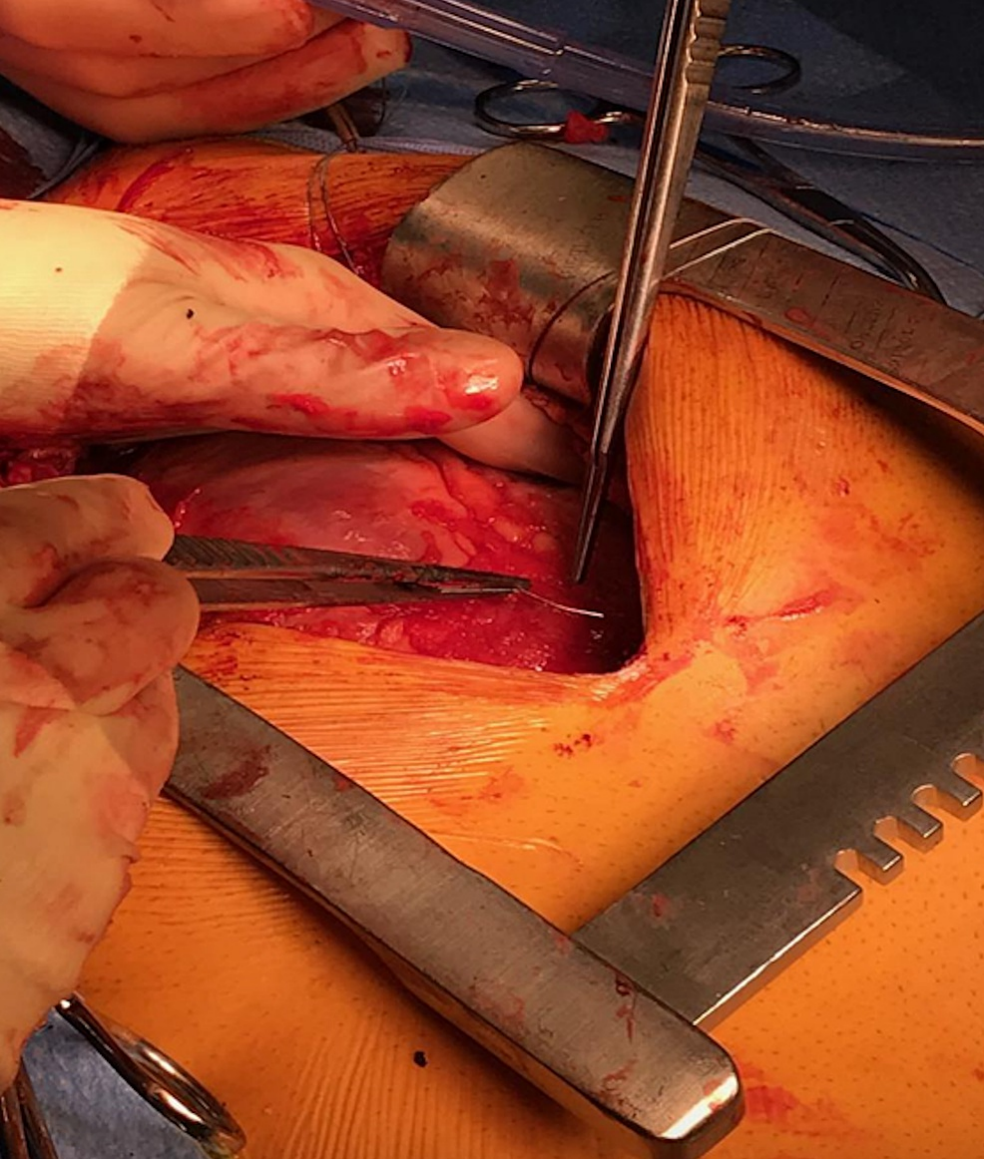
Needle during open sternotomy

Given the sudden hypotension and tachycardia, the patient was given rapid intravenous fluids to temporize his blood pressure while being rushed to the operating room where an emergent median sternotomy was performed. During this procedure, 500 ml of fluid was drained from within the pericardial sac. Upon exploration of the mediastinum, a needle was found in the fibrous scarring of the right ventricular epicardium (Figure 4). The needle had burrowed through the right ventricular cavity to the outer surface of the right ventricular epicardium. A second needle was not found; thus, the pericardial sac was copiously irrigated, three chest tubes were placed, and the sternum was closed. The patient was then placed on cefuroxime for 48 hours based on a concern of possible endocarditis. However, given his normal white blood cell count, lack of fever, improving chest pain, and negative blood cultures at 48 hours, an infectious disease consultant approved the cessation of antibiotic therapy. CT of the right upper extremity and chest were obtained to confirm that there was not a second needle in the heart.

The right upper extremity CT revealed a needle within the antecubital fossa, which was stable and not damaging nearby structures. It was therefore decided to not remove the needle and to observe the patient on an outpatient basis. The three chest tubes drained an additional 500 ml of fluid before being removed, and the patient was discharged with close follow-up on post-operative day 4 after an uneventful post-operative course. The patient was seen a month later and had no recurrence of pain and no post-operative complications. Unfortunately, the patient presented to the emergency department again within 45 days of the original admission for methamphetamine overdose. He was again stabilized with supportive care and discharged.

## Discussion

Needle fragment embolization occurs in IVDUs when a piece of an intravenous needle breaks off and escapes into the systemic vasculature. In a retrospective study of 70 IVDUs conducted by Norfolk and Gray, 20% of the subjects reported a history of broken needle fragments during drug use [1]. Although there are multiple reports of needle embolization related to various etiologies, including acupuncture, self-inflicted injury, and trauma, needle fragment embolization to the heart in the IVDU population has rarely been reported [2,3]. After an extensive literature search, we found only 12 case reports describing cardiac embolization of needle fragments in IVDUs between 1988 and 2019 [3-14]. The characteristics of these case reports, which included the chief complaint, cardiac complications, and the type of intervention performed, are noted in Table 1.

**Table 1:** Clinical characteristics of case reports of cardiac needle embolizations IJ - Internal jugular; RV - Right ventricle; LV - Left ventricle

Author	Year	Chief complaint	Pericarditis/Pericardial effusion	EKG changes	Location of embolization	Type of intervention performed
Horattas and Moorman [4]	1988	No cardiac symptoms	No	Not mentioned	Right atrium/ventricle	None
Gyrtrup et al. [5]	1989	Chest pain	Yes	Not mentioned	Right ventricle wall	Thoracotomy/pericardial fluid drained. Needle removed
Lemaire et al. [6]	1998	Chest pain	Abscess under pericardium	Diffuse t wave inversions	Right ventricle wall in the abscess	Open sternotomy-abscess drained. Needle removed
Thorne and Collins [7]	1998	Incidental finding at autopsy	No	No	Right ventricle wall	None. Incidental finding at autopsy after sudden death.
Ngaage and Cowen [8]	2001	Dyspnea	No	No	Right ventricle wall	Not suitable for transvenous removal. Patient refused surgical intervention.
Low et al. [9]	2005	Chest pain	No	Widespread concave ST elevation	Right ventricle inferior wall	As there is no effusion/stable hemodynamically. Treated conservatively with analgesics.
Steiner et al. [10]	2012	Chest pain	Yes	No EKG changes seen	Right ventricle	Pericardiocentesis/pericardial window placement for recurrent effusion. Needle remove with a bioptome/through right IJ.
Al-Sahaf et al. [11]	2016	Chest pain/dyspnea	Yes	None mentioned	Right ventricle septum	Pericardiocentesis. Thoracotomy needle removal one year later as needle migrated and caused pneumothorax.
Danek et al. [3]	2016	Chest pain	No	Peaked T waves indicating early repolarization	RV apex myocardium/projecting into LV	Stable hemodynamically, no intervention. Stable at one year follow-up.
Bensted et al. [12]	2017	Chest pain	Yes	None mentioned	Intraventricular septum--migrated to pericardial space	Sternotomy- drainage and removal of needle.
Hill et al. [13]	2019	Chest pain	Yes	ST elevation-inferior/lateral leads	RV pericardial space	Pericardiocentesis. Effusion was not re-occurring. So conservative approach. Needle not removed.
Yen et al. [14]	2019	Chest pain	Yes	None mentioned	RV, penetrating the wall. Embedded in the myocardium	Pericardiocentesis. Endovascular approach to remove needle unsuccessful. So conservative approach favoured over surgery. Two months follow-up echo stable.

Considering the number of IVDUs and reported frequency of broken needle fragments by this population, the low number of case reports of needle embolization to the heart in this population is surprising. This could be due to multiple factors, for example, the reluctance of this population to seek medical attention and misdiagnoses given the asymptomatic nature in many cases (Table 1). In IVDUs presenting to the health care setting with symptoms such as chest pain and dyspnea, special attention must be paid to the possibility of needle embolization to the heart.

Here, we present a very rare case of accidental needle loss in a patient’s arm, which then embolized to his myocardium and pericardium through his venous system. Even more surprising is the fact that this patient did not suffer from cardiac infection after the introduction of the foreign body. However, as noted in similar case reports (Table 1), these needles can be surprisingly well tolerated without symptoms for years. They therefore do not always need to be removed as long as the patient is asymptomatic, there is no ongoing damage to the surrounding structures, and the patient is followed up closely with imaging provided on an as-needed basis. There are currently no guidelines for the treatment of such patients, and failure to remove the needle can lead to complications such as cardiac tamponade, infections, embolisms, or arrhythmias, which need to be considered during medical decision-making regarding surgery for these patients. In the present case, the patient was unstable with signs of cardiac strain, severe angina, and dyspnea. It was therefore decided that the benefits of exploratory sternotomy outweighed the risks. The patient tolerated the procedure well with good outcomes and the removal of the needle from his heart. Thus, it is the authors’ opinion that close consideration of the risks and benefits of major surgical procedures should be considered in such patients, and they should be treated in a multidisciplinary manner with close follow-up that includes consultations with specialists in cardiovascular surgery, cardiology, infectious disease, internal medicine, psychiatry, wound care, and drug abuse rehabilitation, as necessary.

## Conclusions

This case study illustrates the importance of including needle embolization to the heart as a potential differential diagnosis in patients with past or current drug abuse presenting with angina and/or dyspnea. It further highlights the lack of relevant guidelines available, key considerations in withholding treatment in asymptomatic patients, and treatment considerations in symptomatic patients (e.g., possible cardiac tamponade, infection, embolism, and arrhythmia), including sternotomy with exploration of the mediastinal cavity. Working with a multidisciplinary team is essential for good outcomes in these patients, and treatment must be tailored in a patient-specific manner. Shared decision-making with the patient and discussions regarding the risks and benefits of treatment vs. watchful waiting are recommended for these patients.
